# Changes in lipid composition of host-derived extracellular vesicles following *Salmonella* infection

**DOI:** 10.1128/spectrum.02796-23

**Published:** 2023-12-11

**Authors:** Lisa E. Emerson, Chanel A. Mosby, Samantha Enslow, Winnie W. Hui, Melissa K. Jones, Mariola J. Ferraro

**Affiliations:** 1 Microbiology and Cell Science Department, IFAS, University of Florida, Gainesville, Florida, USA; Universidad Andres Bello, Santiago, Chile

**Keywords:** exosomes, *Salmonella*, bacterial infection, lipidomics, extracellular vesicles, lipids

## Abstract

Extracellular vesicles are small membrane-bound structures secreted by host cells that are involved in a variety of functions and the transmission of cargo. Vesicular cargo contains a variety of biomolecules, including lipids. Limited research has focused on the cargo content of extracellular vesicles during infections, particularly the alterations in lipid cargo that occur following bacterial infections. Our study investigates the lipid composition of extracellular vesicles generated by RAW 264.7 macrophages before and after infection with *Salmonella enterica* serovar Typhimurium at 24 and 48 hours post-infection. Our results reveal that both time and infection conditions significantly alter specific lipids in host-derived small extracellular vesicles. The altered lipids primarily comprise glycerophospholipids, sphingolipids, and prenol lipids. Our findings have important implications for understanding molecular mechanisms underlying the trafficking of lipids to extracellular compartments during infection.

**IMPORTANCE:**

This study delves into the previously unexplored territory of extracellular vesicle (EV) cargo and composition, specifically focusing on lipid composition changes in EVs following *Salmonella* infection. EVs play crucial roles in intercellular communication, carrying a variety of biomolecules. Investigating how these EV cargo lipids change post-infection with *Salmonella* is significant for understanding the molecular mechanisms underlying lipid trafficking during infection. Given the impact of lipid composition on EV function, this research uncovers distinct differences in the lipid profiles of EVs at different time points post-infection and between infected and uninfected macrophages. This study identified lipids that are differentially abundant in EVs produced by the host during infection, offering novel insights into the dynamics of lipid profiles in EVs during cellular processes and infections. This work advances our understanding of host-pathogen interactions, specifically lipid-mediated EV functions during infection.

## INTRODUCTION

Extracellular vesicles (EVs) are small membrane-bound structures secreted by host cells (1) that play essential functions in intracellular communication and the transmission of various biomolecules. Exosomes are a specific type of small EV (sEV) that are 30–150 nm in diameter and are formed endosomal sorting complex required for transport (ESCRT) dependent and independent as endosomes mature into multivesicular bodies and form intraluminal vesicles. These vesicles then fuse with the plasma membrane and release exosomes into the extracellular space (2). The sEV cargo varies based on the cell of origin (3, 4) and may contain proteins, metabolites, nucleic acids, and lipids (5). The sEV cargo is known to mediate host-pathogen interactions (6 – 8), cell-to-cell communication (9), and host-immune responses (10, 11).

Recent studies have examined the lipid composition in sEVs isolated from mouse oligodendroglial cells (12), human B cells (13), mast cells RBL-2HS (14), dendritic cells (14), and in-vitro guinea pig reticulocytes (15). Changes in EV lipid content have been reported in cancer and Parkinson’s disease (16, 17). Previous studies comparing EVs to total cell membranes have found that certain lipids, such as sphingomyelin, phosphatidylserine, as well as ceramides (12 – 14, 18), are enriched in EVs. In addition, two sEV biogenesis pathways are known: the aforementioned ESCRT-dependent process and an ESCRT-independent process utilize ceramides, suggesting that we can expect ceramides to be increased in abundance in sEV fractions as compared to cell membranes (6). However, to date, no studies have investigated the lipid content of sEVs before and after bacterial infection. Considering the significant role that lipid composition plays in the sEV function, we have conducted a study to investigate the lipid content of sEVs released from RAW 264.7 macrophages infected with *Salmonella*. Specifically, our study aimed to characterize the lipid composition of sEVs at 24 and 48 hours post-infection (hpi) with *Salmonella enterica* serovar Typhimurium and to highlight the differences in lipid composition between sEVs from infected vs uninfected macrophages.

## RESULTS

### The sEV lipid composition shows distinction by both time point and infection

**Fig 1:**
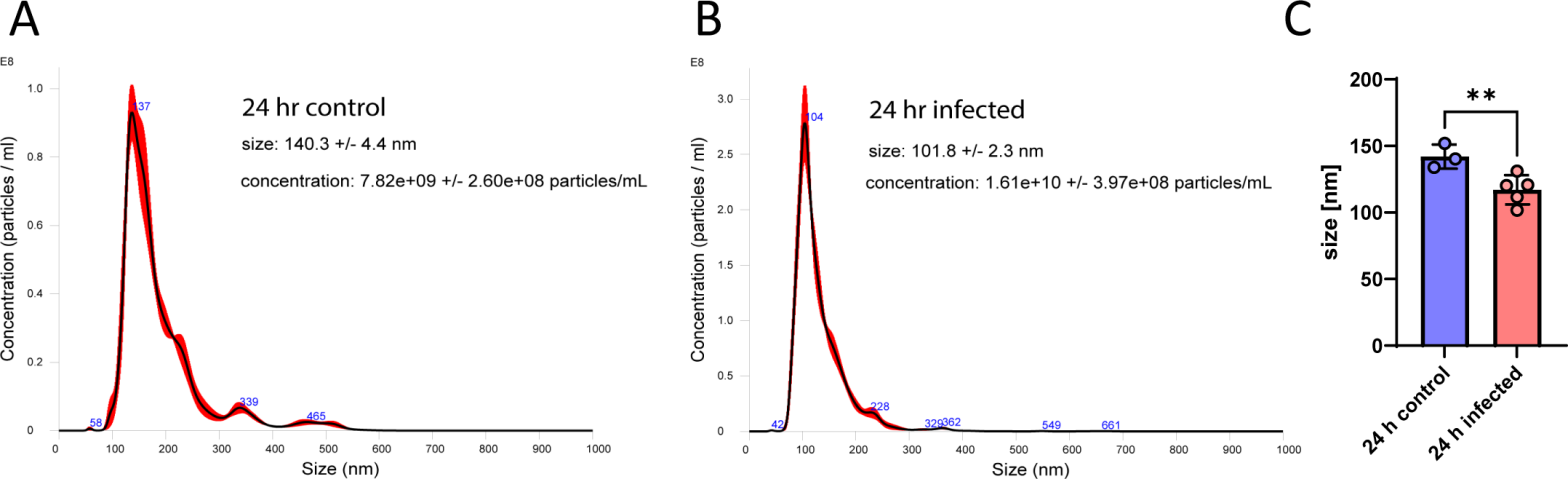
Size and the concentrations of isolated sEVs. Size and concentrations of small extracellular vesicles (sEVs) isolated from RAW 264.7 cells cultured for 24 hours (**A**) or infected with *Salmonella* Typhimurium for 24 hours (**B**). The size and concentrations of the EVs at 24 hpi were determined using nanotracking analysis conducted by Nanosight. (**C**) Change in sEV size between uninfected and Salmonella-infected macrophages at 24 hours post-infection. The analysis involved multiple biological replicates (*n* ≥ 3), and statistical significance was determined using an unpaired *t*-test (*P* value = 0.0167).

The study aimed to determine changes in the lipid profile of sEV fractions before and after *Salmonella* infection. Small EV fractions were isolated at 24 and 48 hours after infections as previously described (19), with sEVs collected from either infected (MOI 5:1) or uninfected treatment conditions. The cytotoxicity of *Salmonella*-infected vs uninfected cells was measured and not shown to be significant (Fig. S1). The sizes and concentrations of the obtained EVs were calculated by using nanoparticle tracking analysis (NTA) (Fig. 1; Fig. S2). The size of EVs displayed notable variations before and after infection. In the case of 24-hour infected samples, EVs exhibited a reduced size in comparison to their counterparts in 24-hour uninfected samples. Quantitative analysis of these EVs, based on data obtained from multiple biological replicates of EVs isolated at the 24-hour infection time point, demonstrated a statistically significant reduction in vesicle size in infected EVs (Fig. 1C). A similar size trend was observed at the 48-hour time point, with EVs from infected samples displaying smaller dimensions than those from uninfected samples. Mass spectrometry analysis was performed on the sEV fractions, and the positive and negative ion modes were separately analyzed to identify lipid species. Initial filtering resulted in the identification of 231 features in the positive and negative ion modes across all conditions. Principal component analysis (PCA) was conducted to analyze the lipid profiles, and distinct clustering patterns based on treatment condition and timepoint were observed (Fig. S3). Furthermore, a heatmap visualization (Fig. S4) demonstrated clear separation of the samples based on both timepoint and treatment condition, with the most significant distinction observed between the 24 and 48 hour time points, followed by grouping based on infection condition.

Additionally, we have conducted a comprehensive analysis of the lipid composition in sEVs produced by RAW 264.7 cells to identify the relative abundances of the identified lipids for each experimental condition, without comparisons to other groups (Fig. S5). For sEVs derived from control cells cultured for 24 hours, most lipids belonged to the glycerolipid category. This category was followed by fatty acyls, prenol lipids, sphingolipids, and glycerophospholipids. In control sEVs from the 48-hour culture, the composition was a little different, as there were slight changes in the lipid proportions, particularly with an increase in sphingolipids and glycerophospholipids compared to other lipid categories. In contrast, sEVs derived from infected cells at 24 hours hpi showed a notable decrease in glycerophospholipids compared to other categories. However, in sEVs derived from 48 hpi, there was an increase in the proportion of sphingolipids and glycerophospholipids, accompanied by a decrease in glycerolipids compared to the other lipid categories (Fig. S5). These observations emphasize the distinct lipid composition of sEVs under different experimental conditions. The changes in lipid categories and their relative abundances provide valuable insights into the dynamics of sEV lipid profiles in response to cellular processes and infections.

### The sEV lipid composition differences due to infection seen at 24 and 48 hour time points

**Fig 2:**
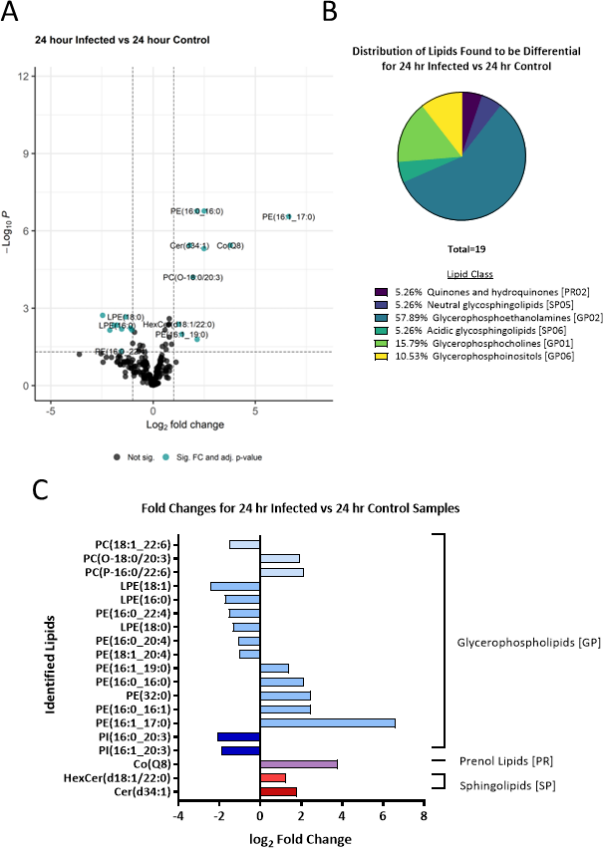
Differential lipid distribution in sEVs generated at 24 hours post-infection with *Salmonella* compared to sEVs from uninfected cells. (**A**) Volcano plot showing the log_2_-fold changes of sEV lipids from RAW 264.7 macrophages infected with *Salmonella* at 24 hpi compared to the 24-hour control condition (uninfected). Fold change values and adjusted *P*-values were calculated using the LipidR R package. A log_2_-fold change cutoff of ±1 and an adjusted *P*-value cutoff of <0.05 was used to determine significance. Significant lipids are indicated in teal and lipids not found to be significant are indicated in black. (**B**) Pie chart presenting the lipid class distribution of the lipids found to be differentially abundant between the infected and control/uninfected conditions at 24 hpi. (**C**) Log_2_-fold changes of the differentially abundant lipids detected between the infected and uninfected/control conditions at 24 hpi. Negative fold changes indicate a decrease in lipid abundance, while positive fold changes indicate an increase. The bars are color-coded based on lipid class, with the corresponding lipid categories indicated on the right-hand side of the graph.

The first comparison investigated was that of infected vs control sEVs at the 24-hour time point. The R package, lipidr, was used to test for differentially abundant lipids using a log_2_-fold change cutoff of ±1 and an adjusted *P* value cutoff of <0.05 (Fig. 2; Table 1; Table S1). This analysis revealed 19 lipids that were found to be differently abundant in sEVs from the 24-hour infection samples vs the 24-hour control samples (Fig. 2B and C), with 10 lipids that had increased abundance and 9 lipids that showed a decrease in abundance. Of these 19 lipids, over half of them belong to the glycerophosphoethanolamines (GP02) class which is part of the glycerophospholipid (GP) category.

**TABLE 1:** The *P*-values and fold changes of features found to be differentially abundant in sEVs from the 24-hour infected vs 24-hour control comparison (*n* = 3)

24-hour infected vs 24-hour control			*P* value	Adjusted *P*-value
Putative molecule annotation	Lipid class abbreviation	log_2_ FC		
LPE(18:1)	LPE	−2.46302	6.88E-05	0.001884197
PI(16:0_20:3)	PI	−2.1116	0.000648	0.007094255
PI(16:1_20:3)	PI	−1.91256	0.000314	0.004585352
LPE(16:0)	LPE	−1.75062	0.000292	0.004564821
PE(16:0_22:4)	PE	−1.54366	0.007088	0.046376145
PC(18:1_22:6)	PC	−1.5359	0.000502	0.006465427
LPE(18:0)	LPE	−1.34073	8.89E-05	0.002163562
PE(16:0_20:4)	PE	−1.09854	0.00043	0.005883134
PE(18:1_20:4)	PE	−1.03532	0.000585	0.006990417
HexCer(d18:1/22:0)	HexCer	1.259114	0.000229	0.004172474
PE(16:1_19:0)	PE	1.403191	0.001026	0.010214836
Cer(d34:1)	Cer	1.782969	8.34E−08	3.65E−06
PC(O-18:0/20:3)	PC	1.949185	2.01E−06	6.28E−05
PE(16:0_16:0)	PE	2.14381	1.55E−09	1.70E−07
PC(P-16:0/22:6)	PC	2.152119	0.001768	0.016137361
PE(32:0)	PE	2.49308	1.34E−07	4.91E−06
PE(16:0_16:1)	PE	2.495075	1.20E−09	1.70E−07
Co(Q8)	CoQ	3.779124	7.24E−08	3.65E−06
PE(16:1_17:0)	PE	6.628221	3.83E−09	2.79E−07

Among the lipids that were more abundant in sEVs after infection of mother cells at 24 hpi, several noteworthy ones emerged. These included Ceramide [Cer(d34:1)] and Hexosylceramide [HexCer(d18:1/22:0)], which both belong to the category of sphingolipids. Additionally, Phosphatidylethanolamine [PE(16:1_19:0), PE(32:0), PE(16:0_16:0), PE(16:0_16:1), and PE(16:1_17:0)] were found to be increased. Furthermore, Phosphatidylcholine [PC(O-18:0/20:3) and PC(P-16:0/22:6)] and Coenzyme Q8 [Co(Q8)], also known as ubiquinone-8, were identified as upregulated lipids. Coenzyme Q8 is a lipid-soluble compound that functions as an electron carrier in the electron transport chain during cellular respiration. In contrast, the lipids downregulated in sEVs following the 24-hour infection of mother cells included Lysophosphatidylethanolamine [LPE(18:1), LPE(18:0), and LPE(16:0)], Phosphatidylinositol [PI(16:0_20:3) and PI(16:1_20:3)], Phosphatidylethanolamine [PE(16:0_22:4), PE(18:1_20:4), and PE(16:0_20:4)], and Phosphatidylcholine [PC(18:1_22:6)], a phospholipid.

**Fig 3:**
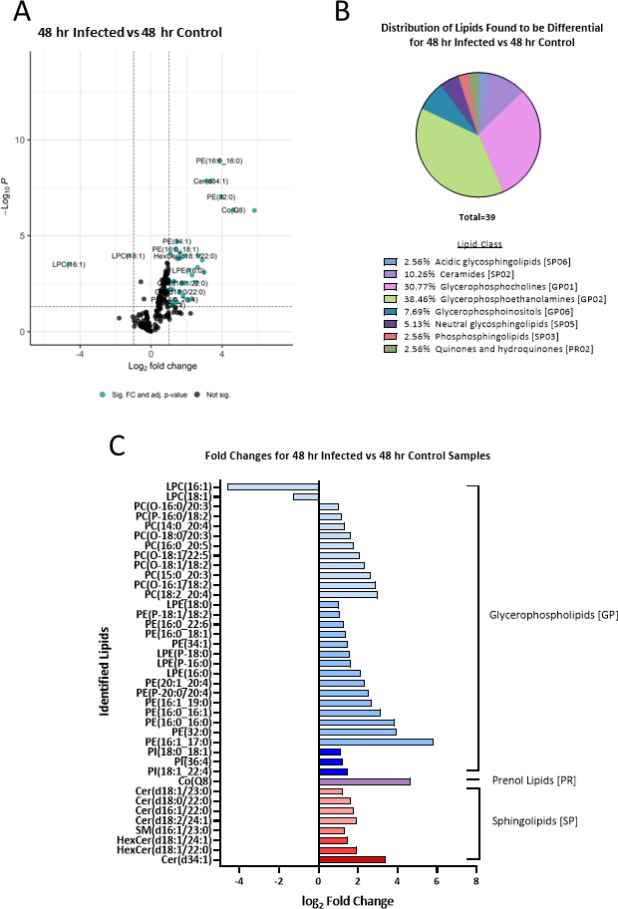
Lipid distribution in sEVs derived from RAW 264.7 macrophages infected with *Salmonella* at 48 hours post-infection compared to sEVs from uninfected cells. (**A**) Volcano plot showing the log_2_-fold changes of sEV lipids from RAW 264.7 macrophages infected with *Salmonella* at 48 hpi compared to the 48-hour control condition (uninfected). Fold change values and adjusted *P* -values were calculated using the LipidR R package. A log_2_-fold change cutoff of ±1 and an adjusted *P*-value cutoff of <0.05 were used to determine significance. Significant lipids are indicated in teal, and lipids not found to be significant are indicated in black. (**B**) Pie chart showing the lipid class distribution of the lipids found to be differentially abundant between the infected and control/uninfected conditions 48 hpi. (**C**) Log_2_-fold changes of the differentially abundant lipids detected between the infected and uninfected/control conditions at 48 hpi. Negative fold changes indicate a decrease in lipid abundance, and a positive fold-change indicates an increase in abundance. The bars are colored by the lipid class with the lipid categories that each lipid falls under indicated on the right-hand side of the graph.

Differentially abundant lipids were also seen when looking at the comparison of the infected vs control sEVs at the 48-hour time point. Using the same log_2_-fold change and adjusted *P* value cutoffs, 39 lipids were found to be significantly differentially abundant with 37 having an increased abundance and 2 lipids showing a decrease in abundance (Fig. 3; Table 2; Table S1). 38.46% of the differential lipids are in the glycerophosphoethanolamines (GP02) class followed by 30.77% being part of the glycerophosphocholines (GP01) class, both classes being part of the glycerophospholipid (GP) category (Fig. 3B).

**TABLE 2:** The *P*-values and fold changes of features found to be differentially abundant in sEVs from the 48-hour infected vs 48-hour control comparison (*n* = 3)

48-hour infected vs 48-hour control			*P* value	Adjusted *P*-value
Putative molecule annotation	Lipid class abbreviation	Log_2_ FC		
LPC(16:1)	LPC	−4.63836	2.79E−05	0.000305139
LPC(18:1)	LPC	−1.2824	5.89E−06	0.000109803
LPE(18:0)	LPE	1.010667	0.00076	0.002972956
PC(O-16:0/20:3)	PC	1.03201	0.022665	0.042424142
PE(P-18:1/18:2)	PE	1.05773	0.001854	0.006345244
PI(18:0_18:1)	PI	1.110719	4.29E−06	9.40E−05
PC(P-16:0/18:2)	PC	1.178303	0.013109	0.028145217
Cer(d18:1/23:0)	Cer	1.202392	0.001845	0.006345244
PI(36:4)	PI	1.214578	0.022047	0.04198514
PE(16:0_22:6)	PE	1.266887	0.000511	0.002434236
SM(d16:1/23:0)	SM	1.306884	0.000506	0.002434236
PC(14:0_20:4)	PC	1.318021	0.00868	0.020889474
PE(16:0_18:1)	PE	1.379128	1.82E−06	4.99E−05
PE(34:1)	PE	1.454302	6.23E−07	1.95E−05
PI(18:1_22:4)	PI	1.46794	0.01372	0.028891067
HexCer(d18:1/24:1)	HexCer	1.469619	1.12E−05	0.000158831
LPE(P-18:0)	LPEP	1.55398	1.16E−05	0.000158831
LPE(P-16:0)	LPEP	1.624974	3.03E−06	7.38E−05
Cer(d18:0/22:0)	Cer	1.640151	0.002697	0.00834661
PC(O-18:0/20:3)	PC	1.643219	9.07E−06	0.000141836
Cer(d16:1/22:0)	Cer	1.774311	0.000721	0.002924042
PC(16:0_20:5)	PC	1.798446	0.005015	0.013901603
Cer(d18:2/24:1)	Cer	1.916569	0.000685	0.002829715
HexCer(d18:1/22:0)	HexCer	1.932131	6.65E−06	0.000112094
PC(O-18:1/22:5)	PC	2.096835	0.009842	0.022688513
LPE(16:0)	LPE	2.102211	6.93E−05	0.00063241
PC(O-18:1/18:2)	PC	2.303846	0.00787	0.01958456
PE(20:1_20:4)	PE	2.306127	0.000161	0.00113524
PE(P-20:0/20:4)	PE	2.521446	0.000622	0.002672772
PC(15:0_20:3)	PC	2.619483	4.27E−05	0.000445364
PE(16:1_19:0)	PE	2.674107	6.02E−06	0.000109803
PC(O-16:1/18:2)	PC	2.885987	1.44E−05	0.00018525
PC(18:2_20:4)	PC	2.996037	9.76E−05	0.000821887
PE(16:0_16:1)	PE	3.1364	1.32E−10	1.40E−08
Cer(d34:1)	Cer	3.372681	1.92E−10	1.40E−08
PE(16:0_16:0)	PE	3.841755	5.53E−12	1.21E−09
PE(32:0)	PE	3.954942	1.71E−09	9.34E−08
Co(Q8)	CoQ	4.64641	1.03E−08	4.51E−07
PE(16:1_17:0)	PE	5.823882	1.31E−08	4.79E−07

The lipids with increased abundance included various sphingolipids such as Sphingomyelin [SM(d16:1/23:0)], Ceramide [Cer(d18:2/24:1), Cer(d16:1/22:0), Cer(d18:0/22:0), Cer(d18:1/23:0), and Cer(d34:1)], and Hexosylceramide [HexCer(d18:1/22:0) and HexCer(d18:1/24:1)]. Additionally, several phospholipids were more abundant in sEVs, including Phosphatidylinositol [PI(18:1_22:4), PI(36:4), and PI(18:0_18:1)], Lysophosphatidylethanolamine [LPE(16:0), LPE(P-16:0), LPE(P-18:0), and LPE(18:0)], and Phosphatidylcholine [PC(18:2_20:4), PC(O-16:1/18:2), PC(15:0_20:3), PC(O-18:1/18:2), PC(14:0_20:4), PC(P-16:0/18:2), PC(O-16:0/20:3), PC(O-18:0/20:3), PC(16:0_20:5), and PC(O-18:1/22:5)]. Furthermore, Phosphatidylethanolamine [PE(32:0), PE(16:1_17:0), PE(16:0_16:0), PE(16:0_16:1), PE(20:1_20:4), PE(P-20:0/20:4), PE(16:1_19:0), PE(34:1), PE(16:0_18:1), PE(16:0_22:6), and PE(P-18:1/18:2)] and Coenzyme Q8 [Co(Q8)], also known as ubiquinone-8, were among the lipids that showed increased abundance in sEVs. On the other hand, the downregulated lipids in sEVs at 48 hpi included Lysophosphatidylcholine (LPC) LPC(16:1) and LPC(18:1) (Fig. 3C).

### The sEV lipid composition in sEVs from infected cells changes over time

**Fig 4:**
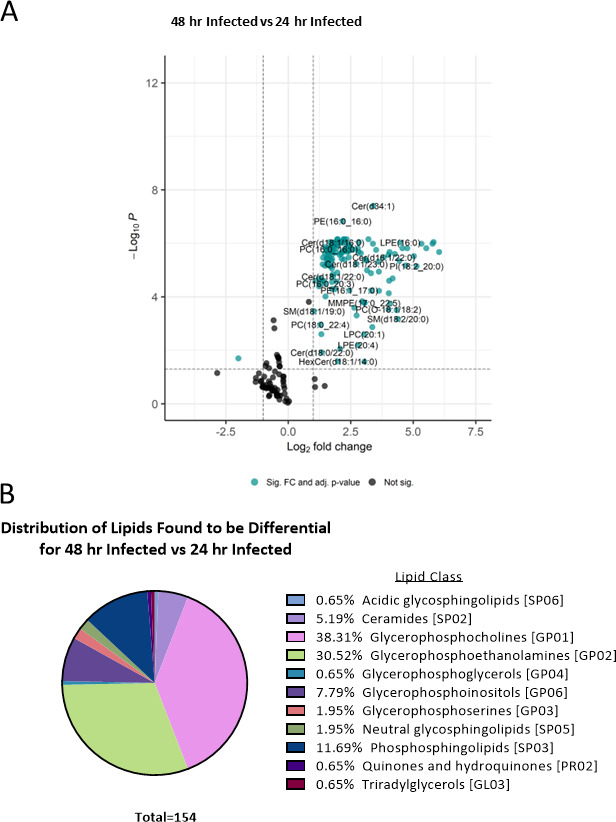
Changes of lipids in sEVs derived from RAW 264.7 macrophages infected or uninfected with *Salmonella* at 24 and 48 hours post-infection. (**A**) Volcano plot showing the log_2_-fold changes of sEV lipids from RAW 264.7 macrophages infected with *Salmonella* at 24 hpi compared to 48 hpi. Fold change values and adjusted *P*-values were calculated using the LipidR R package. A log_2_-fold change cutoff of ±1 and an adjusted *P*-value cutoff of <0.05 were used to determine significance. Significant lipids are indicated in teal and lipids not found to be significant are indicated in black. (**B**) Lipids found to be differential between 48 and 24 hpi via *t*-test with an alpha level of *P* < 0.05.

Furthermore, when comparing the EV-associated lipids from macrophages infected over 48 and 24 hours, the analysis revealed an interesting pattern. To start with, many more lipids were found to be differentially abundant compared to the infected vs control sEVs with a total of 154 lipids identified. Out of these 154 differentially abundant lipids, only one lipid, oxidized triacylglycerol OxTG [14:0_16:0_9:0(COOH)], demonstrated a decrease in abundance (Fig. 4; Table 3; Table S1). This finding suggests that there are dynamic changes in the lipid composition of sEVs over time during the infection process as many other lipids including Ceramides (d34:1, d18:1/16:0, d16:1/22:0) and Sphingomyelinases (d18:1/19:0, d18:2/20:0) significantly increased in abundance in sEVs as the infection of mother cells has progressed. Similar to the distribution of lipid classes seen before, lipids in the glycerophospholipid (GP) category account for the highest percentages of differential lipids with 38.31% of the lipids being part of the glycerophosphocholines (GP01) class and 30.52% being part of the glycerophosphoethanolamines (GP02) class.

**TABLE 3:** The *P* values and fold changes of features found to be differentially abundant in sEVs from the 48-hour infected vs 24-hour infected comparison (*n* = 3)

48-hour infected vs 24-hour infected			*P* value	Adjusted *P*-value
Putative molecule annotation	Lipid class abbreviation	Log_2_ FC		
OxTG(14:0_16:0_9:0(COOH))	OxTG	−1.99626	0.014444	0.019894524
SM(d18:1/19:0)	SM	1.032586	0.000223	0.000341235
PG(18:1_18:1)	PG	1.091702	1.38E−05	2.50E−05
PC(18:0_20:3)	PC	1.227804	2.34E−05	4.04E−05
SM(d17:1/16:0)	SM	1.231703	1.09E−05	2.04E−05
PC(18:0_22:4)	PC	1.282469	0.000736	0.001097164
PE(P-18:0/20:4)	PE	1.295924	1.83E−05	3.20E−05
PC(18:0_22:5)	PC	1.32216	0.00171	0.002497269
PC(16:0_20:4)	PC	1.33953	1.23E−05	2.27E−05
Cer(d18:0/22:0)	Cer	1.351784	0.008472	0.012048329
PC(18:0_20:4)	PC	1.379146	2.87E−06	6.04E−06
SM(d18:1/18:0)	SM	1.394173	1.38E−06	3.41E−06
PC(17:1_17:1)	PC	1.41742	1.38E−05	2.50E−05
SM(d18:1/24:1)	SM	1.461958	8.09E−07	2.35E−06
PC(16:0_22:5)	PC	1.476341	3.33E−06	6.87E−06
SM(d18:2/16:0)	SM	1.479258	2.39E−07	1.38E−06
PC(16:0_20:3)	PC	1.481748	1.84E−05	3.20E−05
PI(18:0_20:4)	PI	1.490685	5.90E−05	9.64E−05
PC(O-16:0/22:5)	PC	1.524663	1.54E−06	3.74E−06
SM(d16:1/22:0)	SM	1.524855	3.62E−07	1.62E−06
SM(d18:1/22:0)	SM	1.538838	6.34E−07	2.05E−06
PE(P-16:0/20:4)	PE	1.547987	2.80E−06	5.95E−06
PE(P-16:0/22:6)	PE	1.552608	1.37E−06	3.41E−06
PC(18:0_22:6)	PC	1.562956	2.11E−06	4.67E−06
PC(O-16:0/18:1)	PC	1.567158	4.47E−07	1.72E−06
SM(d18:1/24:0)	SM	1.568394	3.91E−07	1.65E−06
PS(18:0_18:1)	PS	1.571129	2.58E−06	5.59E−06
PC(16:0_22:6)	PC	1.572387	4.20E−07	1.70E−06
PC(O-18:0/16:0)	PC	1.574514	6.78E−07	2.09E−06
PE(O-16:0/22:6)	PE	1.578414	1.60E−06	3.80E−06
PE(P-18:0/22:5)	PE	1.591592	1.24E−06	3.22E−06
PC(16:0_18:1)	PC	1.606867	5.25E−07	1.92E−06
PC(O-16:1/18:0)	PC	1.616029	6.03E−07	2.05E−06
PC(16:0_16:0)	PC	1.623012	3.90E−07	1.65E−06
SM(d18:1/16:0)	SM	1.631973	3.18E−07	1.52E−06
SM(d17:1/24:1)	SM	1.633845	4.08E−07	1.69E−06
SM(d18:2/22:0)	SM	1.654847	4.38E−07	1.71E−06
PC(O-16:0/16:0)	PC	1.664982	1.38E−06	3.41E−06
PC(14:0_16:0)	PC	1.668889	2.59E−07	1.45E−06
Cer(d18:1/24:0)	Cer	1.670989	6.34E−08	8.98E−07
HexCer(d18:1/24:1)	HexCer	1.678303	3.52E−06	7.13E−06
SM(d17:1/24:0)	SM	1.685942	6.53E−07	2.05E−06
PE(P-18:0/20:3)	PE	1.699753	6.40E−07	2.05E−06
PE(P-18:0/22:6)	PE	1.704681	3.55E−06	7.14E−06
PC(18:0_18:1)	PC	1.710516	2.64E−07	1.45E−06
PI(18:0_20:3)	PI	1.717258	1.94E−07	1.23E−06
PC(16:0_16:1)	PC	1.729931	1.96E−07	1.23E−06
SM(d16:1/16:0)	SM	1.732521	2.26E−07	1.38E−06
PE(P-18:1/22:6)	PE	1.739105	1.98E−06	4.47E−06
PE(18:0_20:4)	PE	1.748685	4.39E−06	8.73E−06
HexCer(d18:1/22:0)	HexCer	1.748832	1.58E−05	2.78E−05
PC(O-16:0/20:4)	PC	1.752473	2.98E−05	5.11E−05
PC(16:0_22:4)	PC	1.752738	1.65E−06	3.88E−06
PE(P-18:0/18:1)	PE	1.758599	3.55E−07	1.62E−06
PC(12:0_16:0)	PC	1.76763	9.20E−07	2.52E−06
PI(34:1)	PI	1.780484	7.64E−06	1.46E−05
PE(P-16:0/20:3)	PE	1.781067	1.28E−07	1.04E−06
PE(P-16:0/18:1)	PE	1.783399	1.83E−07	1.21E−06
Cer(d18:1/22:0)	Cer	1.788603	9.28E−06	1.75E−05
Cer(d18:1/16:0)	Cer	1.788615	7.82E−08	9.27E−07
SM(d18:2/24:1)	SM	1.79186	5.52E−07	1.93E−06
PC(18:1_18:1)	PC	1.792621	2.38E−07	1.38E−06
PC(16:0_18:2)	PC	1.848405	8.36E−08	9.27E−07
SM(d18:1/18:1)	SM	1.855426	1.02E−07	1.04E−06
PE(16:0_18:1)	PE	1.861882	1.16E−07	1.04E−06
SM(d16:1/23:0)	SM	1.867593	3.03E−05	5.15E−05
PE(18:0_18:1)	PE	1.878283	1.66E−07	1.21E−06
LPE(P-16:0)	LPEP	1.899254	7.44E−07	2.20E−06
PC(18:0_20:2)	PC	1.901743	5.51E−08	8.98E−07
PE(34:1)	PE	1.916939	4.81E−08	8.98E−07
PE(18:0_22:6)	PE	1.919615	3.26E−07	1.52E−06
PE(P-18:1/18:1)	PE	1.923284	1.75E−07	1.21E−06
PE(P-18:0/20:5)	PE	1.929891	1.50E−05	2.67E−05
PE(16:0_16:1)	PE	1.960353	1.20E−08	7.01E−07
PC(17:0_18:1)	PC	1.962519	6.38E−06	1.24E−05
PI(18:0_18:1)	PI	1.979402	2.12E−08	7.01E−07
HexCer(d18:1/14:0)	HexCer	1.991875	0.019118	0.026004877
PC(15:0_18:1)	PC	1.998256	1.42E−07	1.11E−06
PC(O-16:0/18:2)	PC	2.036659	5.42E−08	8.98E−07
LPE(P-18:0)	LPEP	2.052492	9.85E−07	2.66E−06
PC(18:2_18:2)	PC	2.074706	0.006072	0.008690817
PI(18:1_20:2)	PI	2.100546	8.31E−07	2.35E−06
PC(P-16:1/18:0)	PC	2.11084	6.94E−08	8.98E−07
PE(P-16:0/18:2)	PE	2.131307	8.36E−07	2.35E−06
PE(P-20:0/18:2)	PE	2.132891	1.56E−06	3.75E−06
PE(18:1_18:1)	PE	2.148078	6.97E−08	8.98E−07
PE(16:0_16:0)	PE	2.176152	1.34E−09	1.47E−07
PC(O-18:0/20:3)	PC	2.205725	6.56E−07	2.05E−06
PC(14:0_18:2)	PC	2.215419	1.32E−08	7.01E−07
PE(16:1_18:1)	PE	2.217763	4.65E−07	1.76E−06
Cer(d18:1/24:1)	Cer	2.231316	2.56E−08	7.01E−07
PI(18:1_20:4)	PI	2.245871	1.72E−06	4.00E−06
PC(16:0_18:3)	PC	2.282498	1.21E−07	1.04E−06
PE(P-16:0/16:1)	PE	2.314364	4.54E−08	8.98E−07
PI(18:1_18:1)	PI	2.336453	1.64E−08	7.01E−07
PE(18:1_20:4)	PE	2.351036	6.15E−07	2.05E−06
PE(18:0_22:4)	PE	2.36065	3.02E−07	1.52E−06
LPE(18:0)	LPE	2.412963	5.43E−07	1.93E−06
PE(16:0_20:4)	PE	2.417912	5.56E−07	1.93E−06
PC(O-16:0/22:4)	PC	2.419534	1.33E−06	3.39E−06
PE(32:0)	PE	2.421191	1.76E−07	1.21E−06
PE(16:1_17:0)	PE	2.442811	3.39E−05	5.72E−05
PC(16:1_18:2)	PC	2.462126	1.74E−07	1.21E−06
PE(18:1_20:2)	PE	2.479691	3.84E−07	1.65E−06
PC(17:0_18:2)	PC	2.598467	4.38E−07	1.71E−06
PS(18:1_18:1)	PS	2.642024	0.000163	0.00025463
Cer(d18:1/23:0)	Cer	2.730141	2.75E−06	5.91E−06
PC(P-16:0/22:6)	PC	2.732666	0.000326	0.000495401
PC(17:1_20:3)	PC	2.789876	3.07E−07	1.52E−06
LPE(20:4)	LPE	2.791718	0.004393	0.006329149
PE(16:0_22:6)	PE	2.809783	6.44E−07	2.05E−06
PC(20:2_20:4)	PC	2.823297	5.02E−08	8.98E−07
Co(Q8)	CoQ	2.904278	8.25E−07	2.35E−06
PC(O-16:0/22:6)	PC	2.958038	9.02E−05	0.000146248
LPC(20:1)	LPC	3.036815	0.001738	0.00252087
SM(d18:2/17:0)	SM	3.042081	0.019608	0.02650753
MMPE(17:0_22:5)	MMPE	3.04322	0.000105	0.000167666
PI(18:1_22:4)	PI	3.055373	0.000106	0.000168835
PE(16:1_19:0)	PE	3.07557	1.74E−06	4.00E−06
PS(O-18:0/17:0)	PSO	3.136582	1.95E−06	4.45E−06
PI(16:1_20:3)	PI	3.150848	5.15E−06	1.02E−05
PC(18:1_20:4)	PC	3.206307	2.49E−08	7.01E−07
PC(O-16:0/20:3)	PC	3.309168	6.79E−06	1.30E−05
PE(18:0_20:3)	PE	3.318939	3.49E−05	5.83E−05
PC(O-18:0/18:2)	PC	3.364141	0.000919	0.001359156
PE(P-18:1/18:2)	PE	3.388874	1.19E−07	1.04E−06
Cer(d34:1)	Cer	3.393525	1.81E−10	3.96E−08
PC(18:1_22:6)	PC	3.512724	4.75E−07	1.76E−06
PC(P-18:1/18:1)	PC	3.607624	2.23E−06	4.87E−06
PC(18:0_20:1)	PC	3.621616	5.84E−06	1.14E−05
PC(O-16:1/20:4)	PC	3.632132	3.61E−05	6.00E−05
Cer(d16:1/22:0)	Cer	3.821872	1.29E−06	3.33E−06
LPE(18:1)	LPE	4.009797	1.01E−06	2.71E−06
PC(O-18:1/18:2)	PC	4.012921	0.000189	0.000294177
PC(P-18:1/20:4)	PC	4.025861	4.48E−05	7.37E−05
PE(18:1_18:2)	PE	4.035276	1.40E−05	2.50E−05
PC(O-16:1/18:2)	PC	4.054218	7.05E−07	2.12E−06
PC(O-18:1/22:5)	PC	4.071659	0.000111	0.000175387
PE(P-20:0/20:4)	PE	4.17457	1.11E−05	2.05E−05
Cer(d18:2/24:1)	Cer	4.266434	8.99E−07	2.49E−06
PE(P-18:1/20:5)	PE	4.337747	0.000205	0.000316421
SM(d18:2/20:0)	SM	4.392128	0.000442	0.000668172
PI(36:4)	PI	4.443614	2.00E−06	4.47E−06
LPE(16:0)	LPE	4.483807	8.47E−08	9.27E−07
PC(15:0_20:3)	PC	4.566867	3.18E−07	1.52E−06
PC(16:0_20:5)	PC	4.706533	3.23E−06	6.74E−06
PC(P-16:0/18:2)	PC	4.763424	3.05E−07	1.52E−06
PC(18:2_20:4)	PC	5.029123	1.14E−06	3.00E−06
PI(18:2_20:0)	PI	5.135571	3.49E−06	7.13E−06
PE(20:1_20:4)	PE	5.32916	1.07E−07	1.04E−06
PE(16:0_22:4)	PE	5.522559	3.27E−07	1.52E−06
PI(16:0_20:3)	PI	5.770409	1.27E−07	1.04E−06
PC(14:0_20:4)	PC	5.814731	6.55E−08	8.98E−07
PC(16:1_20:4)	PC	6.034963	7.00E−07	2.12E−06

### The sEV lipid composition in sEVs from uninfected cells over time

**Fig 5:**
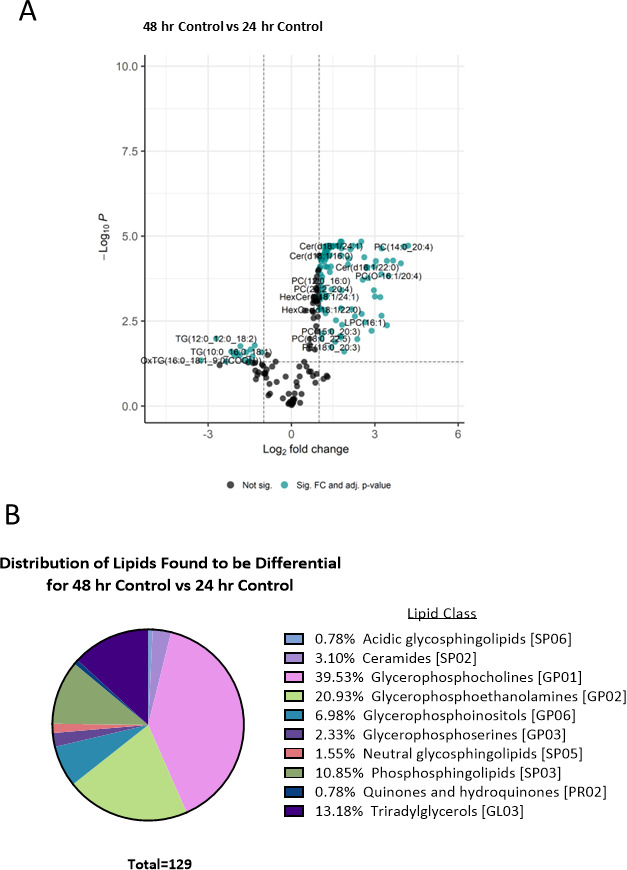
Changes of lipids in sEVs derived from RAW 264.7 uninfected macrophages at 24 and 48 hours of culture. (**A**) Log_2_-fold changes of lipids in sEVs derived from RAW 264.7 uninfected macrophages at 24 and 48 hours of culture. Significant lipids determined via *t*-test with a *P*-value of *P* < 0.05 are indicated in teal and non-significant results indicated in black. (**B**) Differential lipids between the 48 and 24 hour control conditions determined via *t*-test with an alpha level of *P* < 0.05.

To investigate the variances in lipid abundance in sEVs generated from cells growing over a period of time but without the presence of *Salmonella* infection (sEVs derived from uninfected cells), we conducted a comprehensive lipid analysis in cells incubated over the 48 and 24 hour (Fig. 5; Table 4; Table S1). The result was that a similarly high number of differentially abundant lipids was seen in the comparison of 48 vs 24 hour control sEVs with 129 lipids identified. Of these 129 lipids, 112 showed increased abundance and 17 showed a decrease in abundance. Interestingly, all 17 of the decreased lipids consisted of oxidized triacylglycerol and triacylglycerol (Table 4; Table S1). The distribution of the lipids is similar to that seen with the infected 48 vs infected 24 hour where the two highest percentages are part of the glycerophospholipid (GP) category: 39.53% of the lipids in the glycerophosphocholines (GP01) class and 20.93% are in the glycerophosphoethanolamines (GP02) class.

**TABLE 4:** The *P*-values and fold changes of features found to be differentially abundant in sEVs from the 48-hour control vs 24-hour control comparison (*n* = 3)

48-hour control vs 24-hour control			*P* value	Adjusted *P*-value
Putative molecule annotation	Lipid class abbreviation	Log_2_ FC		
OxTG(16:0_18:1_9:0(COOH))	OxTG	−3.25015	0.032178	0.045172372
TG(12:0_12:0_18:2)	TG	−2.70915	0.006264	0.010313612
TG(18:1_18:2_22:0)	TG	−2.34319	0.03495	0.048443431
TG(10:0_16:0_18:1)	TG	−2.16646	0.016707	0.02506047
TG(16:0_18:1_8:0)	TG	−2.04129	0.022633	0.032396652
TG(17:0_17:1_17:1)	TG	−2.00005	0.01827	0.026853495
TG(16:0_18:1_18:1)	TG	−1.95136	0.019031	0.027785013
TG(12:0_16:0_18:1)	TG	−1.85709	0.021892	0.031542149
TG(16:0_18:1_18:2)	TG	−1.83479	0.011125	0.017402096
TG(12:0_14:0_16:0)	TG	−1.70996	0.031745	0.044852316
TG(16:0_16:0_18:2)	TG	−1.62879	0.017674	0.026188765
TG(16:0_16:0_18:1)	TG	−1.50734	0.034396	0.047978522
TG(10:0_14:0_16:0)	TG	−1.49593	0.036355	0.049761309
TG(12:0_16:0_18:2)	TG	−1.46954	0.014285	0.021724963
TG(16:0_16:1_16:1)	TG	−1.3962	0.024071	0.034231121
TG(16:0_18:2_18:2)	TG	−1.31992	0.010501	0.016545021
TG(18:1_18:1_18:2)	TG	−1.05991	0.017698	0.026188765
HexCer(d18:1/24:1)	HexCer	1.001804	0.000257	0.000603686
PE(18:1_20:4)	PE	1.007759	0.00071	0.001416996
PE(18:1_20:2)	PE	1.025316	0.000637	0.001303262
PC(16:0_16:0)	PC	1.027418	2.25E−05	7.91E−05
PC(16:0_18:1)	PC	1.027471	2.72E−05	8.82E−05
PE(18:0_22:4)	PE	1.037711	0.000335	0.000733134
SM(d16:1/22:0)	SM	1.043673	1.08E−05	4.59E−05
PC(15:0_18:1)	PC	1.048553	4.33E−05	0.000131602
PC(12:0_16:0)	PC	1.063823	7.44E−05	0.000204717
PC(18:0_22:5)	PC	1.070632	0.006313	0.010316784
Cer(d18:1/16:0)	Cer	1.075304	7.92E−06	3.77E−05
HexCer(d18:1/22:0)	HexCer	1.075815	0.000733	0.001446346
PC(16:0_18:2)	PC	1.078923	1.09E−05	4.59E−05
SM(d18:1/22:0)	SM	1.080596	1.46E−05	5.52E−05
PC(17:1_17:1)	PC	1.094768	0.000117	0.000312626
PI(18:1_20:2)	PI	1.104682	0.000198	0.000482396
PC(18:0_20:3)	PC	1.108406	5.49E−05	0.000158291
PC(18:1_22:6)	PC	1.110414	0.004385	0.007386475
PC(20:2_20:4)	PC	1.150005	0.000137	0.000356359
PC(O-16:0/18:2)	PC	1.16924	8.42E−06	3.84E−05
SM(d18:1/16:0)	SM	1.169544	6.40E−06	3.59E−05
SM(d18:2/16:0)	SM	1.171394	2.01E−06	2.06E−05
SM(d17:1/24:1)	SM	1.189295	7.07E−06	3.70E−05
SM(d17:1/24:0)	SM	1.193483	1.40E−05	5.42E−05
SM(d18:1/24:1)	SM	1.199248	4.77E−06	2.90E−05
PC(18:1_18:1)	PC	1.209776	8.22E−06	3.83E−05
PC(17:0_18:2)	PC	1.218229	0.000279	0.000624337
PS(18:0_18:1)	PS	1.221018	2.31E−05	7.91E−05
PE(18:0_18:1)	PE	1.225511	7.84E−06	3.77E−05
PI(18:1_18:1)	PI	1.229203	5.98E−06	3.45E−05
PC(18:0_22:4)	PC	1.229698	0.00099	0.001869076
SM(d16:1/23:0)	SM	1.232129	0.000773	0.001511884
PI(18:0_20:3)	PI	1.235984	3.85E−06	2.48E−05
PC(17:0_18:1)	PC	1.240554	0.000278	0.000624337
PE(P-18:0/20:3)	PE	1.243506	1.04E−05	4.54E−05
SM(d18:1/24:0)	SM	1.249577	3.06E−06	2.32E−05
PC(18:0_18:1)	PC	1.250007	4.51E−06	2.82E−05
PI(18:1_20:4)	PI	1.251897	0.000236	0.000567096
PE(34:1)	PE	1.255913	2.35E−06	2.06E−05
SM(d18:2/22:0)	SM	1.262522	5.01E−06	2.96E−05
PC(16:0_22:6)	PC	1.26346	3.04E−06	2.32E−05
PE(16:0_18:1)	PE	1.269184	3.83E−06	2.48E−05
SM(d16:1/16:0)	SM	1.278224	3.58E−06	2.46E−05
PC(16:0_22:4)	PC	1.290211	2.40E−05	8.09E−05
PE(16:0_16:1)	PE	1.319028	4.86E−07	1.92E−05
PI(18:0_18:1)	PI	1.321199	9.06E−07	1.92E−05
PC(O-16:1/18:0)	PC	1.332263	3.44E−06	2.46E−05
PC(14:0_18:2)	PC	1.335738	1.45E−06	1.95E−05
SM(d18:2/24:1)	SM	1.348447	7.04E−06	3.70E−05
PE(P-16:0/20:3)	PE	1.354033	1.58E−06	1.95E−05
PC(O-16:0/18:1)	PC	1.361613	1.61E−06	1.95E−05
PC(18:0_20:2)	PC	1.365593	1.17E−06	1.95E−05
PC(O-16:0/16:0)	PC	1.381433	7.27E−06	3.70E−05
PE(P-16:0/18:2)	PE	1.381634	3.71E−05	0.000114485
PE(P-16:0/18:1)	PE	1.407662	1.59E−06	1.95E−05
PC(15:0_20:3)	PC	1.42134	0.003702	0.006333613
PC(16:1_18:2)	PC	1.424406	2.31E−05	7.91E−05
PC(18:0_20:4)	PC	1.424446	2.15E−06	2.06E−05
PE(P-18:0/18:1)	PE	1.427894	2.36E−06	2.06E−05
Cer(d18:1/24:1)	Cer	1.429949	1.57E−06	1.95E−05
PE(18:0_20:3)	PE	1.430034	0.011898	0.018479881
PC(O-16:0/20:4)	PC	1.443615	0.000144	0.000371915
PC(P-16:1/18:0)	PC	1.449599	2.19E−06	2.06E−05
PE(P-18:1/18:1)	PE	1.486359	1.84E−06	2.04E−05
PC(O-18:0/16:0)	PC	1.52422	9.12E−07	1.92E−05
PC(18:2_20:4)	PC	1.575524	0.008028	0.012832568
LPE(18:1)	LPE	1.603546	0.001676	0.003008395
PE(18:1_18:1)	PE	1.646886	8.13E−07	1.92E−05
PE(P-16:0/16:1)	PE	1.685908	8.54E−07	1.92E−05
PC(18:0_22:6)	PC	1.750051	7.61E−07	1.92E−05
PI(18:1_22:4)	PI	1.750482	0.00524	0.008759385
SM(d18:1/18:1)	SM	1.766583	1.62E−07	1.45E−05
PE(18:1_18:2)	PE	1.779663	0.005774	0.009579194
PC(O-16:1/18:2)	PC	1.78363	0.000657	0.001332106
PE(P-20:0/18:2)	PE	1.797347	7.12E−06	3.70E−05
PE(16:1_18:1)	PE	1.80022	3.07E−06	2.32E−05
Cer(d34:1)	Cer	1.803813	7.48E−08	1.45E−05
PE(16:1_19:0)	PE	1.804653	0.00016	0.000396948
PS(18:1_18:1)	PS	1.823926	0.002316	0.00408962
PC(O-18:1/22:5)	PC	1.897758	0.016418	0.024796733
Co(Q8)	CoQ	2.036993	1.89E−05	6.90E−05
PE(P-18:1/18:2)	PE	2.049525	1.11E−05	4.60E−05
PC(17:1_20:3)	PC	2.125986	3.59E−06	2.46E−05
PI(36:4)	PI	2.169424	0.000712	0.001416996
PC(18:1_20:4)	PC	2.22383	7.48E−07	1.92E−05
PE(P-20:0/20:4)	PE	2.281804	0.001255	0.002309066
PC(O-18:1/18:2)	PC	2.369673	0.006719	0.010819972
PC(O-16:0/22:4)	PC	2.417522	1.34E−06	1.95E−05
PC(O-18:0/20:3)	PC	2.511691	1.98E−07	1.45E−05
PI(18:2_20:0)	PI	2.527786	0.001042	0.001950419
PE(20:1_20:4)	PE	2.565472	6.87E−05	0.000192878
PS(O-18:0/17:0)	PSO	2.624468	9.42E−06	4.21E−05
LPC(16:1)	LPC	2.647809	0.001939	0.00345185
PC(P-18:1/18:1)	PC	2.703175	2.74E−05	8.82E−05
Cer(d16:1/22:0)	Cer	2.730262	2.46E−05	8.17E−05
PC(18:0_20:1)	PC	2.753415	5.98E−05	0.000170095
PE(P-18:1/20:5)	PE	2.88413	0.003541	0.006105299
Cer(d18:2/24:1)	Cer	2.965263	2.21E−05	7.91E−05
PC(16:0_20:5)	PC	2.966326	0.000157	0.000394885
PC(16:1_20:4)	PC	3.007711	0.000259	0.000603686
PC(O-16:0/20:3)	PC	3.04135	1.41E−05	5.42E−05
PC(P-18:1/20:4)	PC	3.197129	0.000279	0.000624337
PC(O-16:0/22:6)	PC	3.23132	4.39E−05	0.000131607
PC(O-18:0/18:2)	PC	3.243123	0.001184	0.002197492
PE(16:1_17:0)	PE	3.24715	2.90E−06	2.32E−05
PI(16:0_20:3)	PI	3.434032	1.35E−05	5.39E−05
SM(d18:2/20:0)	SM	3.437284	0.002409	0.004220256
PC(O-16:1/20:4)	PC	3.49463	4.97E−05	0.000147222
PE(16:0_22:4)	PE	3.656612	1.30E−05	5.29E−05
PC(P-16:0/22:6)	PC	3.937369	1.71E−05	6.36E−05
PC(14:0_20:4)	PC	4.039148	1.87E−06	2.04E−05
PC(P-16:0/18:2)	PC	4.200517	9.64E−07	1.92E−05

## DISCUSSION

In this study, we conducted an analysis of the lipidome of sEVs at 24 and 48 hpi with *Salmonella enterica* serovar Typhimurium UK-1.

First, we observed variations in the size of sEVs before and after infection with *Salmonella* at both the 24- and 48-hour time points. The sEVs derived from infected samples exhibited a significant reduction in size compared to those from uninfected samples. The observed reduction in size of sEVs changes in sEV size underscore the dynamic nature of these vesicles during *Salmonella* infection, suggesting that alterations in size could be due to altered biogenesis. Indeed, host proteins that constitute these vesicles during infection represent a significant departure from the protein composition of sEVs in uninfected cells (20). These alterations in protein content and size suggest a potential role of *Salmonella* infection to sEV biogenesis. Lipid arrays within EVs are known to vary based on EV size. A previous study (21) used asymmetric-flow field-flow fractionation to categorize exosomes from various cell types by size: large exosomes (Exo-L; 90–120 nm), small exosomes (Exo-S; 60–80 nm), and a smaller non-membranous group called exomeres (∼35 nm). Phosphatidylcholine was the primary lipid in all exosomes, comprising 46%–89% of the lipid content. Sphingomyelin levels were generally low (2%–10%). Interestingly, some exomers displayed elevated levels of diglycerides and triglycerides. The intriguing observation in our study was the simultaneous decrease in the size of EVs during infection and an increase in specific lipid classes. This points to the need for a more detailed investigation into the vesicle biogenesis processes and their relationship with these lipid changes.

Our results demonstrate that specific lipids in small EVs are significantly altered by both time and infection conditions. However, it is important to acknowledge that the differences in lipids between infected and uninfected EVs at the 24-hour time point appeared relatively modest. This observation likely arises from the dynamic nature of lipid changes over time, implying that more substantial alterations may manifest as the infection progresses. This finding also suggests there is a need for further investigation to comprehensively understand the evolving lipid dynamics in response to infection. Furthermore, it becomes evident that future in-depth analyses are required to enhance the identification of unidentified lipids, which could hold critical clues to the evolving molecular landscape within EV during infection.

A comparison between the 24- and 48-hour infection conditions revealed 154 differentially expressed lipids, with the glycerophosphocholine class being the most prominently affected. These observations underscore the importance of considering control conditions in experimental design to differentiate infection-specific changes since the lipid composition of EVs changes over the time as the cells grow. Next, we performed the comparison between sEVs generated from cells infected with *Salmonella* and uninfected. The analysis of sEV fractions revealed distinct clustering patterns based on treatment condition and timepoint, indicating variations in lipid composition.

Through mass spectrometry analysis, 19 lipids were identified as significantly altered at 24 hours hpi, including sphingolipids and phospholipids. At 48 hpi, a total of 39 differentially expressed lipids were identified, encompassing sphingolipids, phospholipids, and other lipid classes. Notably, glycerophospholipids, particularly glycerophosphocholine and glycerophosphoethanolamines, exhibited significant changes in abundance during the infection process. These findings highlight the remodeling of glycerophospholipids in sEVs during bacterial infection. It is interesting to note that many of these lipids that increased in sEVs derived from infected cells were also identified in uninfected cells [including Cer(d18:2/24:1), Co(Q8), Cer(d34:1), PC(14:0_20:4), PC(15:0_20:3), PC(18:2_20:4), PE(20:1_20:4), PI(36:4), or PC(O-16:1/18:2], so while it is possible that bacteria contribute lipid content of host-derived sEVs during infection, all of the lipids increased in sEVs derived from infected cells were also identified in sEVs from uninfected cells.

Among the critical lipid groups remodeled in sEVs during *Salmonella* infection were glycerophospholipids, which are also the primary lipid component of cell membranes, and sEVs are composed of processed cell membrane components; thus, sEVs can be expected to include glycerophospholipids. However, glycerophospholipids were found to be the most abundant differentially expressed class of lipids, suggesting roles for the glycerophospholipid lipid category in sEVs generated after infection (22).

Spingolipids were one of the classes of lipids associated with EVs increased in infection at both time points. This class of lipids, characterized by their amino-alcohol backbone, were initially recognized as essential components of cell membranes, contributing to the formation of lipid rafts involved in cell signaling. However, recent studies have revealed that many sphingolipids are bioactive molecules that regulate various cellular functions, including inflammation (23), apoptosis (24), and autophagy (25). Ceramides, as bioactive sphingolipids, have been implicated in the regulation of immune responses (26). Despite their structural and functional diversity, sphingolipids share common synthetic and degradation pathways. They can be synthesized *de novo*, derived from the hydrolysis of sphingomyelin by using serine palmitoyl transferase, or generated through the salvage pathway by recovering sphingosine from complex sphingolipids. In all cases, the synthesis of ceramide serves as a crucial step. Moreover, sphingolipids have garnered significant interest in the field of extracellular vesicles (EVs) due to their diverse function (27, 28) (12). In our study, sphingolipids emerged as the third most common lipid category in our samples and were among the three differentially abundant lipid categories following infection. The increased abundance of ceramides in sEVs after bacterial infection implies their potential involvement in modulating host-pathogen interactions, which is highly likely due to the recognized function of these sphingolipids in key host processes. As one example, sphingolipids are already known to be involved in host response to *Salmonella* infection (29). Inhibition of serine palmitoyl transferase, a key enzyme in *de novo* sphingolipid biosynthesis, using the inhibitor myriocin, has been shown to impact the process of autophagy. Specifically, myriocin treatment leads to a decrease in autophagy by activating Akt and suppressing beclin-1 (25). Furthermore, myriocin treatment results in the downregulation of ERK and inhibits the membrane recruitment of NOD2 and ATG16L1. The interaction between ATG16L1 and NOD2 in epithelial cells is responsible for the autophagic degradation of *Salmonella*. Additionally, myriocin reduces the expression of LC3-II, a marker of autophagy (30). These findings indicate that sphingolipids may play a role in facilitating *Salmonella*-induced cellular autophagy of damaged *Salmonella*-containing vacuoles (SCVs) in epithelial cells.

Moreover, the identification of increased proportion of ceramides in the lipid composition of sEVs formed during *Salmonella* infection might have another important implication related to their biogenesis. Exosomes are known to be enriched in cholesterol as well as sphingolipids [specifically sphingomyelin (SM) and hexosylceramide], and the release of these EVs is reduced after the inhibition of neutral sphingomyelinase (N-SMase) (31). We have previously identified that GW4869 inhibitor of N-SMase did reduce the number of CD63-positive EVs (likely exosomes) during *Salmonella* infection of human macrophages (19). An increase in ceramides in the sEVs formed during infection might indicate that these vesicles are exosomes.

Prenol lipids are the third lipid category that was significantly increased in EVs from infected vs control cells. In both infected vs control analyses, coenzyme Q8 Co(Q8) was found to be significantly increased. Coenzyme Q is a quinone cell membrane lipid that has a role in oxidative phosphorylation and electron transport (32). Quinones are well conserved across eukaryotic and prokaryotic cells as membrane lipids and electron transporters (33).

Remarkably, phosphatidylserine was consistently detected (but not differentially altered) in our sEVs (Table S1), demonstrating its presence as a stable component that remained unaltered even under the influence of infection conditions. This lipid has been previously established as a prominent constituent in EVs, notably exhibiting enrichment in macrophage-derived EVs following *Mycobacterium tuberculosis i*nfection (34). Also, phosphatidylserine was found to mediate the effects of these EVs on macrophages, as evidenced by the reversal of these effects upon phosphatidylserine blockade (34).

Our study also revealed an intriguing trend in the abundance of glycerophospholipids, prenol lipids, and specifically sphingolipids, which exhibited increased levels in sEVs during *Salmonella* infection. Sphingolipids, especially ceramide, play a major role in the biogenesis of exosomes. This process involves the ceramide transport protein (CERT), responsible for transporting ceramide from the endoplasmic reticulum (ER) (35) to the multivesicular endosome (MVE) at contact sites (27). CERT’s pleckstrin homology (PH) domain interacts with phosphatidylinositol 4-monophosphate (PI4P) at the MVE, facilitating ceramide translocation. Next, a complex forms between CERT and Tsg101, a component of the endosomal sorting complex required for transport (ESCRT-I), further connecting ceramide to the ESCRT-dependent pathway for EV biogenesis (27). Inhibition of ceramide synthesis reduces the formation of the CERT-Tsg101 complex and diminishes EV production, resulting in lower ceramide and sphingomyelin levels in EVs (27). This underscores the likelihood that the formation of ceramide-rich EVs during *Salmonella* infection is associated with an amplified CERT-mediated process of EV production, which can be tested in the future.

This study is not without its limitations. First and foremost, the sample size was restricted, and this was particularly noticeable in the case of the 24 hour samples (Fig. S2). Additionally, it is important to acknowledge that *Salmonella* infection not only impacted sEV lipid composition but also exerted a broad influence on macrophages. These macrophages, undergoing *Salmonella* infection, were subjected to a state of general stress. This overarching stress condition could potentially affect a wide range of cellular functions that contribute to the modification of sEV lipids. Although no significant toxicity was observed at the 24 hour post-infection mark (Fig. S1), the host cell was undergoing various other stressors as a consequence of the infection. At this juncture, it is not feasible to pinpoint which specific aspect of *Salmonella* pathogenesis had the greatest impact on the alteration of sEV lipid composition. Moreover, our study represents the first investigation into the differences in sEV lipids before and after Gram-negative bacterial infection, and therefore, there was a lack of existing literature on this specific topic to draw more conclusions. However, this pioneering study serves as a foundation for future research aimed at exploring the lipid content of sEVs during bacterial infection.

Overall, this study provides valuable insights into the differential lipid abundance in sEVs during bacterial infection and highlights the significance of lipid remodeling in EVs in the context of host-pathogen interactions. Future research will explore specific EV lipids differentially increased or decreased following infection and their roles in biogenesis, infection, or cell-to-cell communication. Despite the limitations, our study provides an important first look into the lipidome of sEVs before and after bacterial infection and opens the door to further research on the roles of lipids in extracellular vesicles during host-pathogen interactions.

## MATERIALS AND METHODS

All key resources are listed in Table 5.

**TABLE 5:** Key resources

Resource	Source	Identifier	Additional information
RAW264.7 macrophages	ATCC	TIB-71	
*Salmonella enterica* serovar Typhimurium (UK-1)	Gift from Roy Curtiss III Lab	ATCC 68169	
Protease inhibitor cocktail	Pierce	A32963	
Ultracentrifuge tubes	Beckman	344058	Open top 38.5 mL
DMEM media	Gibco	11971-025	High glucose, no phosphate

### Cell culture

RAW 264.7 Macrophages (ATCC, TIB-71) were cultured in Dulbecco’s modified Eagle medium (DMEM, Gibco) supplemented with EV-free 10% fetal bovine serum (FBS) and 1% Penicillin-Streptomycin (Pen-Strep, Gibco). Specifically, any EVs were removed from FBS via ultracentrifugation at 120,000×*g* for 18 hours at 4°C. The cells were maintained at 37°C in a humidified atmosphere with 5% CO_2_ until reaching 80% confluency. Subsequently, they were seeded in T-75 flasks at a density of 2.1 × 10^6 cells prior to *Salmonella* infection.

### Isolation of sEVs

sEVs were isolated as previously described by our group (19, 20, 36), in which publications we established protocols that ensured that EVs have appropriate tetraspanin markers, dontu-like shape, and they lack significant and measurable contamination with other bacterial and host products. Briefly, RAW264.7 macrophages were infected with *Salmonella enterica* serovar Typhimurium UK-1 (ATCC 68169) at an MOI of 5:1 for 2 hours, incubated with Gentamicin at 100 mg/mL for 60 minutes, and media collected at 24 and 48 hpi for isolation of sEVs. The sEVs were also collected from cells cultured in absence of infection as controls. The sEV-containing media were spun at 500×*g*, 4,000×*g*, and 16,000×*g* subsequently to remove debris. Next, the media were filtered through a 0.22-µm filter and then ultracentrifuged at 100,000 for 3 hours. Extracellular vesicle pellets were washed with protease inhibitor cocktail and ultracentrifuged again for 18 hours at 100,000×*g*. The sEV pellets were resuspended in protease inhibitor cocktail at a total volume of 150 µL.

### Nanoparticle tracking analysis

sEVs underwent NTA using the Malvin Nanosight NS300 (UF, ICBR). Post-ultracentrifugation, resuspended sEVs were appropriately diluted in MilliQ H_2_O to allow 20–100 particles per frame of each standard operating procedure (SOP) run. Equipment and SOP configurations were provided by UF ICBR Cytometry Core. Five, 60-second analyses were done for infected and uninfected sEVs collected at 24 and 48 hpi. The replicates were analyzed and summarized collectively. Moreover, for quantification analysis, we also used independent 3–5 biological replicates prepared on separate days.

### Lipid extraction

Global lipidomics profiling was performed by the Southeast Center for Integrated Metabolomics (SECIM) at the University of Florida similarly as we previously published (37). The samples were extracted using 1:2:1 methanol:chloroform:water. The organic layer was subsequently dried down using a steady stream of nitrogen and reconstituted with 30 µL of 2-propanol.

### Mass spectrometry

Global lipidomics profiling was performed using a Thermo Q-Exactive Orbitrap mass spectrometer equipped with a Dionex UHPLC system and autosampler. All samples were subjected to separate injections for analysis in both positive and negative heated electrospray ionization modes. The mass spectrometer operated at a mass resolution of 35,000 at *m*/*z* 200. Separation of lipid species was achieved using an Acquity BEH C18 column (1.7 µm particle size, 100 × 2.1 mm dimensions). The mobile phase composition consisted of two components: mobile phase A, which comprised a mixture of 60% acetonitrile and 40% 10 mM ammonium formate with 0.1% formic acid in water, and mobile phase B, which consisted of a mixture of 90% 2-propanol, 8% acetonitrile, and 2% 10 mM ammonium formate with 0.1% formic acid in water. During analysis, a flow rate of 500 µL/min was maintained, and the column temperature was set to 50°C. For negative ionization, a sample volume of 5 µL was injected, while for positive ionization, 3 µL of the sample was injected.

### Cytotoxicity assay

RAW264.7 cells were plated at a density of 3 × 10^4 in a white-walled 96 well plate 24 hours before *Salmonella* infection. After undergoing the infection protocol described above, cell cytotoxicity was determined using the Promega CellTox Green Cytotoxicity Assay (product #: G8742). CellTox Green Reagent was diluted according to the Endpoint Method protocol provided by Promega. Four microliters of Lysis Solution was added to the appropriate wells 30 minutes prior to incubation with CellTox Green Reagent. Twenty microliters of CellTox Green Reagent was added per well, and cells were incubated for 15 minutes at 37°C in the dark. The absorbance was measured at an excitation of 490 nm and emission of 525 nm using the BioTek Cytation 3 Cell Imaging Multi-Mode Reader.

### Statistical analysis

Lipidomics data analysis was performed as we described in references (37, 37). Briefly, the peak tables for the positive and negative modes were combined before missing values were removed or replaced with the estimated detection limit prior to filtering and normalization by sum on the MetaboAnalyst website (38). In RStudio, the R package “compositions” was then used to transform the data with centered log-ratio (clr) coefficients. The R package “lipidr” was used for principal-component analysis and differential abundance analysis, while the heatmap was generated with the “complex heatmap” R package (39). Statistical significance of the summed abundances for the lipidomics categories and classes was measured using two-way ANOVA with Tukey’s multiple-comparison test. Graphs and plots were made using either GraphPad Prism (version 8.0.0 for Windows, GraphPad Software, San Diego, California USA) or R Studio (version 1.3.1093 with R version 4.0.2). Moreover, while performing the data analysis, to account for the unique lipids associated with bacteria (or bacterial EVs) compared to host cells, we thoroughly analyzed our data sets by comparing them to uninfected cells. By doing so, we ensured that there was no lipid specific to bacteria, as all quantified lipids were also present in EVs from uninfected cells.

## SUPPLEMENTAL MATERIAL

Supplemental figures spectrum.02796-23-s0001.pdf Fig. S1 to S5.

Table S1 spectrum.02796-23-s0002.xlsx
*P* values and fold changes of features in sEVs from macrophages (*n*=3) found by untargeted lipidomics.

ASM does not own the copyrights to Supplemental Material that may be linked to, or accessed through, an article. The authors have granted ASM a non-exclusive, world-wide license to publish the Supplemental Material files. Please contact the corresponding author directly for reuse.
