# Supplementary material for: Changes in lipid composition of host-derived extracellular vesicles following Salmonella infection
Source: Microbiol Spectr. 2023 Dec 11;12(1):e02796-23. doi: 10.1128/spectrum.02796-23 (PMC10783105; doi:10.1128/spectrum.02796-23)
Supplement: Supplemental figures — Fig. S1 to S5. [file spectrum.02796-23-s0001.pdf]

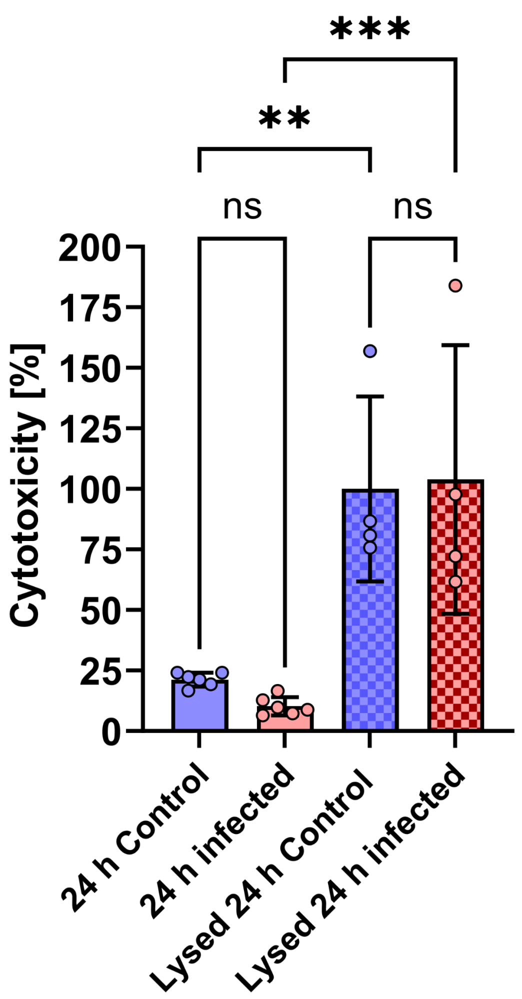

Fig. S1. Cytotoxicity assessment in control and *Salmonella*-infected cells at 24 Hours post-infection. RAW264.7 cells were incubated for 24 hours, followed by infection with *Salmonella*. After a 24-hour incubation period, cells were subjected to one of two conditions: they were either left undisturbed or lysed to release their cellular contents. Cytotoxicity was quantified using the Promega CellTox™ Green Cytotoxicity Assay. Individual biological replicates are represented as data points in the graph and were normalized to the background absorbance readings of the CellTox Green Reagent. Statistical significance was determined through a one-way ANOVA test with Tukey's post-hoc analysis.

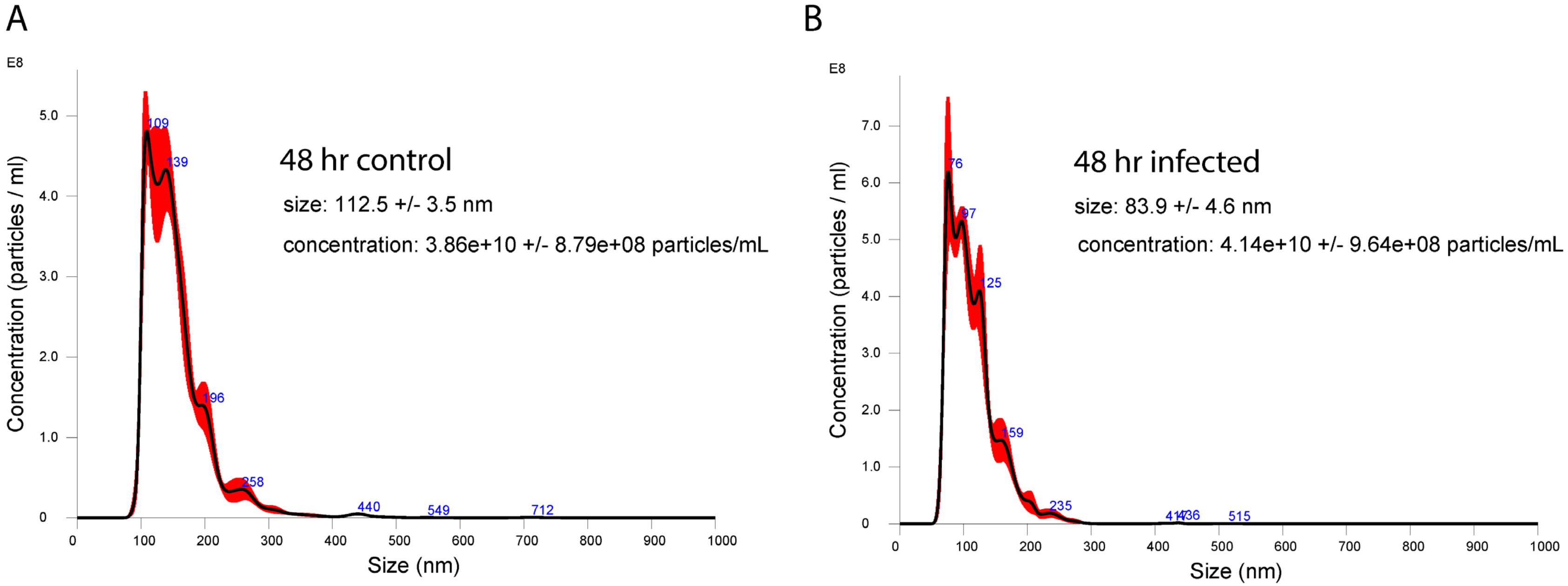

Fig. S2. Size and the concentrations of isolated sEVs. Size and concentrations of extracellular vesicles (EVs) isolated from RAW 264.7 cells cultured for 48 hours **(A)** or infected with *Salmonella* Typhimurium for 48 hours **(B)**. The size and concentrations of the EVs were determined using nanotracking analysis conducted by Nanosight.

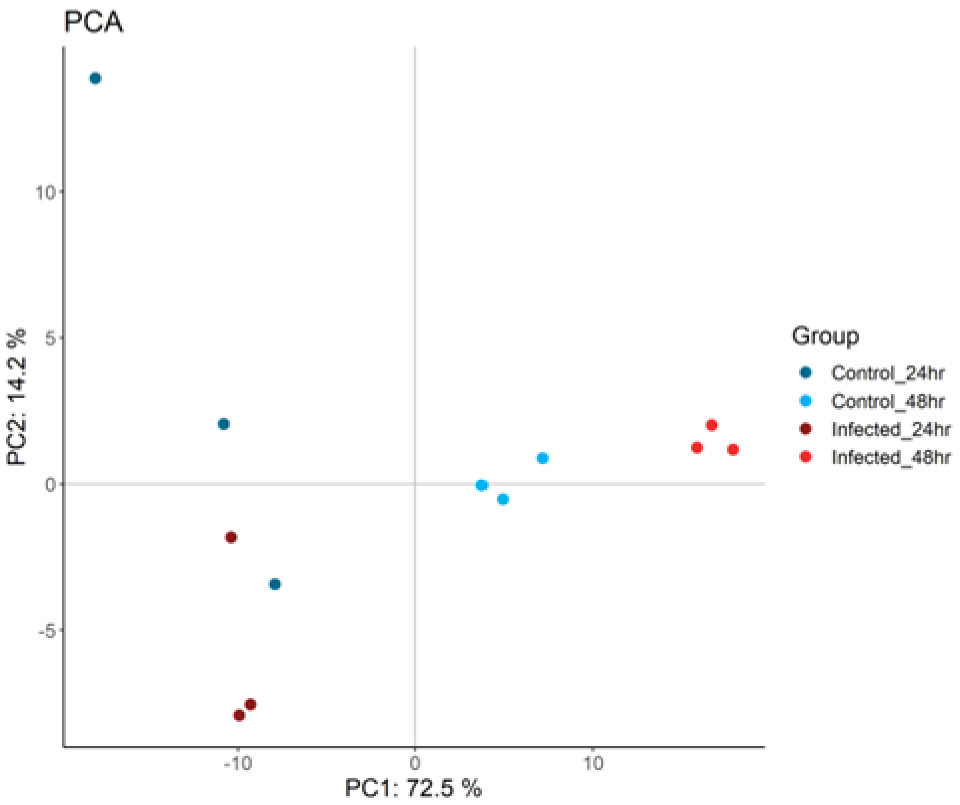

Fig. S3. Global differences in lipid composition of sEVs derived from RAW 264.7 macrophages infected or uninfected with *Salmonella* at 24- and 48-hours post-infection. Principal Component Analysis (PCA) plot shows global differences in lipid composition of sEVs derived from RAW 264.7 macrophages infected or uninfected with *Salmonella enterica* Serovar Typhimurium UK-1 (ATCC 68169) at 24 and 48 hpi. N=3.

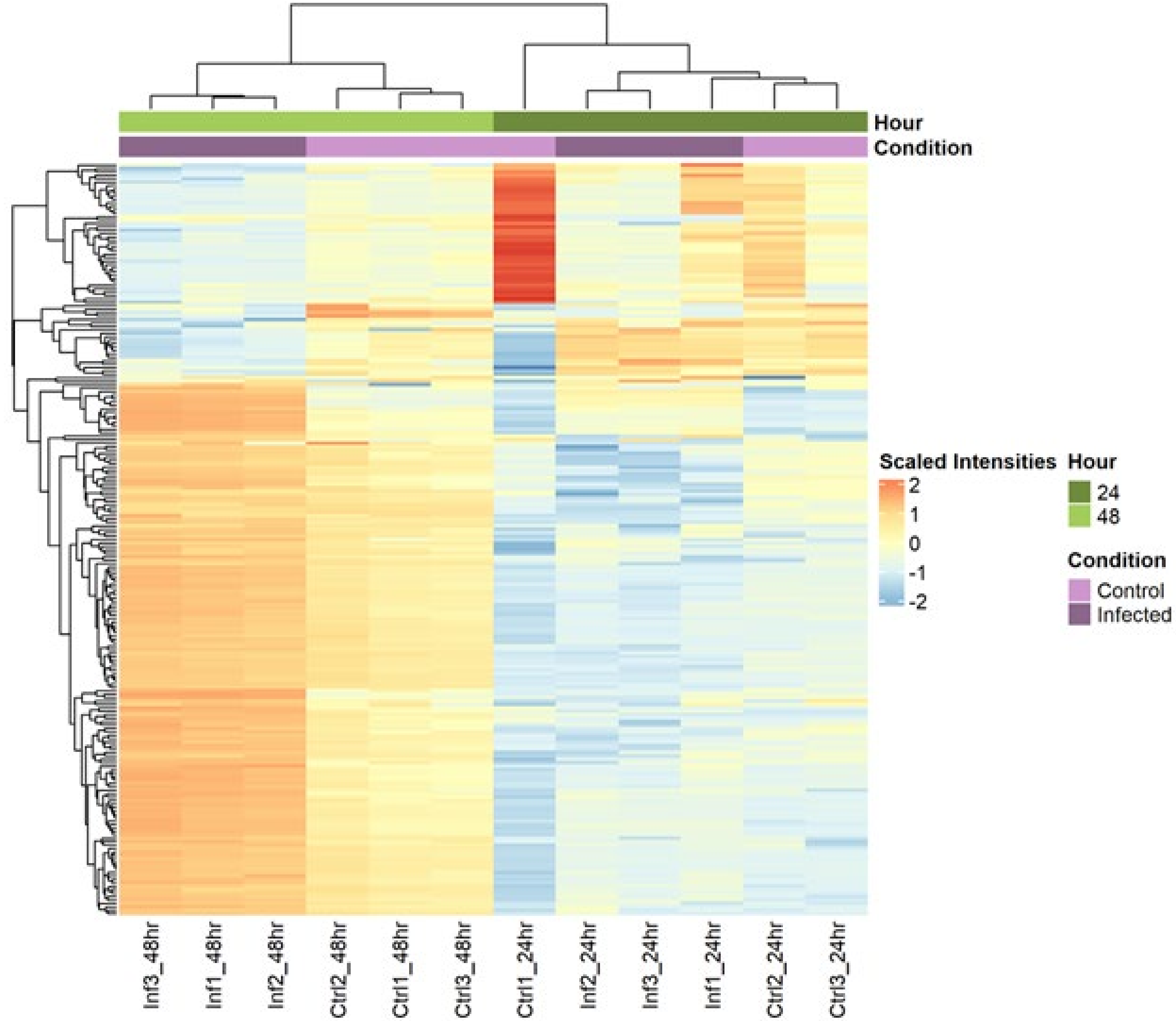

Fig. S4. **Heatmap of normalized and clr-transformed lipidomics results.** Column annotation indicates 24- (dark green) or 48-hour (light green) timepoints and whether each sample is infected (dark purple) or control/uninfected condition (light purple). N=3.

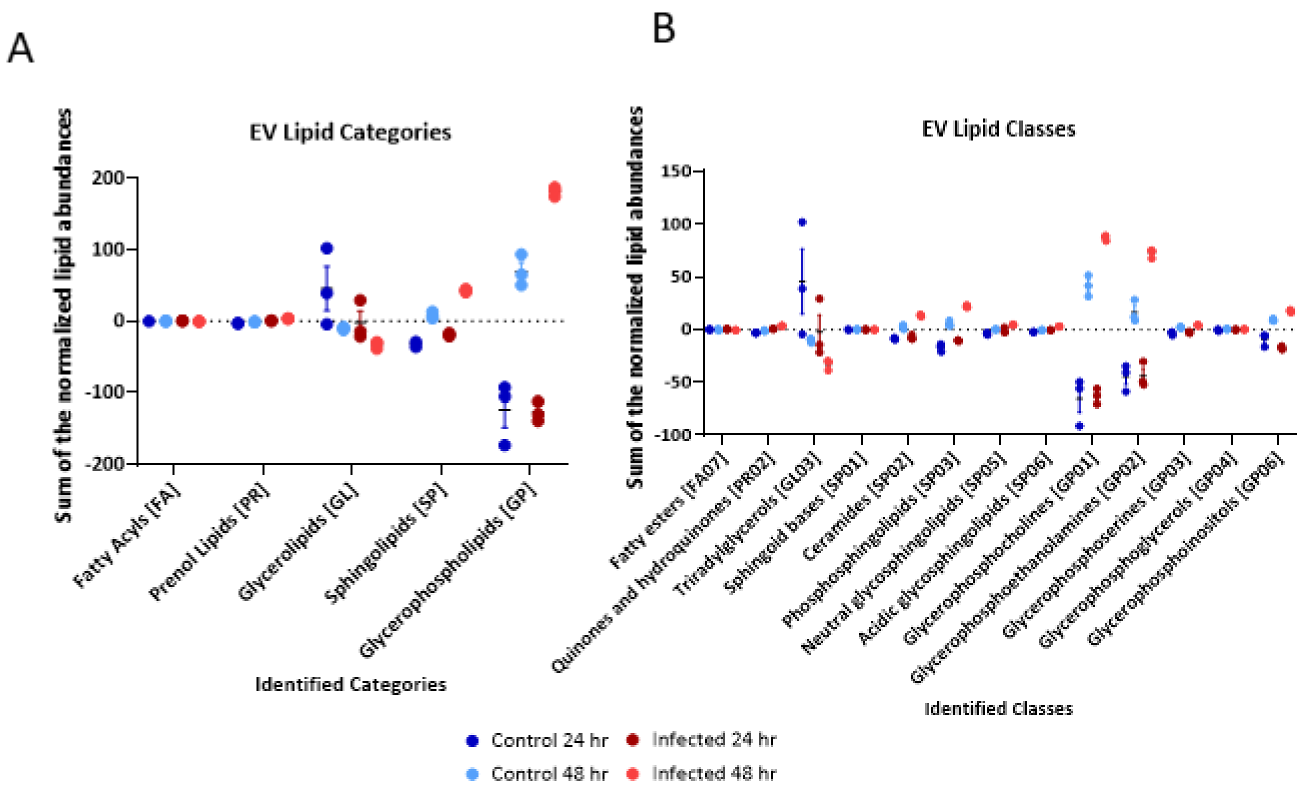

Fig. S5. Class distribution of identified lipids. Lipid categories **(A)** and classes **(B)** identified across all samples (N=24) specified by treatment at both 24 and 48 hpi timepoints using mass spectrometry.
